# Patent-Office Examiners

**Published:** 1890-01

**Authors:** 


### Editorial.

### PATENT-OFFICE EXAMINERS.

Very few persons have any idea of the amount of business done in the Patent-Office in Washington or the technical character of the work necessary to be accomplished in the examination of applications for patents.

Every facility has been provided by the government to assist the examiners in the performance of their duties. Well-equipped laboratories have been placed at their disposal. This is especially noticeable in the chemical and electrical departments. Many of the men are experts in their department. And they must needs be; otherwise the country would be flooded with duplicate patents and the courts overcrowded with "patent" litigation. The examiners are not required to possess a knowledge of outside matters, nor are they expected to know whether an article has been long in use; but, if possessed of such knowledge, they would be capable of rendering much more efficient service to the government. Such knowledge is not always needed, but at times would be most valuable indeed. Professor G. D. Seeley, of the electrical department, is said to be an electrical expert who is thoroughly posted not only in the history of electricity but in its science, and is engaged almost daily in the laboratory testing the practicability of the many inventions that come before him, as well as deciding upon their claims for novelty, etc.

Dr. Antisell is an expert chemist and fully qualified to pass upon inventions that come in his line. Mr. Pierce is also said to be an expert machinist, and especially well posted in the complicated machinery of sewing-machines. And so we might go on through the whole list of examiners and still leave a department which, in our judgment, is not filled as it should be. We refer to dental patents, which form a peculiar class, and should be under the jurisdiction of a competent dentist who is fully conversant with the mechanical features of dentistry. Such a need does not exist in regard to medicine, for there is, strictly speaking, no such thing as a medical patent; but in dentistry, owing to the mechanical nature of its practice and to the fact that the patent system has been fostered by certain dealers and, to a great extent, by the dental press, there is a distinct and large class of patents which have been designated as "dental patents." These affect such a large class of individuals that it seems to us that an examiner should be chosen from the dental ranks who would look out for the large interests at stake in a way that a non-professional man, no matter how competent and faithful, could not do,—some retired practitioner of dentistry, who, through long practice and acquaintance with practical dentistry, would be specially fitted for the position, and no doubt could be readily found to fill the place. With a competent dental examiner in the Patent-Office, it would be impossible for any more patents to be issued upon *methods of practice* which have been in vogue from time immemorial almost,—as some of those which have been issued and passed into the hands of the International Tooth-Crown Company, which, besides the patent on the method of making the so-called "Low bridge," that is still pending in the courts, have patents—according to Dr. Crouse—on "twelve or fifteen different *forms* of crowns," and also on "a *method* of preparing roots for crowning," "for cutting off teeth," "for driving out a pulp," "for filling the end of a root," "for freezing a tooth so as to make it insensible," "for cementing a pin or post into a root," "for filling a tooth with some fibrous material," etc.

Not only would a dental examiner prevent the issue of such patents, but, being one of us, he would look out that abuses in other directions were not foisted upon us, as has been done recently in two instances. The first of these is so well described by "Nemo" in the December number of the *Ohio Journal of Dental Science* that we cannot do better than quote from his article. The case is one where a patent had been granted on the *form* of the gum for artificial teeth. In summing up the law governing the issuing of patents, he says,—

"The plain language of the patent laws of the United States is that the claimant should be the first and original inventor of the thing claimed, also that it should be the result of 'effort, industry, or genius' in the claimant. When these conditions are complied with, no one can reasonably object to the granting of a patent, and the purpose of the law, 'to promote the progress of science and the useful arts,' will be attained.

"Keeping the distinction plainly in view between the purpose of the people in granting patents and that of the inventor in soliciting the grant, we shall see that certain considerations ought not to influence the decisions of the Patent-Office.

"First, the fact that the patents granted by the commissioner may be revised by the courts should not be allowed to influence his decisions. Often, apparently, this is not the case.

"Then, again, too often it seems plain that well-paid solicitors have succeeded in worrying the officials into favorable reports on the most trivial applications for inventions either worthless or requiring a great stretch of the imagination to find anything new or any evidence of 'effort, industry, or genius' in them.

"Such decisions in the Patent-Office afford unscrupulous men an opportunity to hinder the business of other manufacturers or extort royalties to which they have no moral right, although they may legally claim them. . . .

"The patent under consideration, it will be observed, is for a *form* of the artificial gum as connected with artificial teeth.

"Now, the parties who obtained this patent claim to make the most perfect imitations of the natural teeth and gums, and therefore the claim in their patent is for something which is only a copy of a well-known natural form, and not an invention the result of an expenditure of 'effort, industry, or genius,' and therefore, under the law, clearly not patentable.

"Again, suppose the form were patentable, the law requires that the claimant shall be the first and original inventor to entitle him to a patent."

Which was not the case in this instance, as the writer proceeds conclusively to show that Mr. Justi had teeth of similar construction on exhibition at the Centennial Exhibition, and on which he had not asked for any patent, because they were a copy of the natural gums, and therefore not entitled to being patented.

There is another objectionable feature to this patent, and that is that it was allowed to pend from April 6, 1884, till October 15, 1885, when it was finally issued. By allowing the application to lie in the Patent-Office the patentee gained five years additional time in the monopoly, which was finally granted for fourteen years, making nineteen years.

Another similar flagrant case is to be found in the patent granted for an improvement in the foot-rest on the Morrison chair, the application for which was made January 5, 1875, and allowed to lie until August 30, 1887, when it was finally pushed through in time to prevent the free manufacture of said chair. By this means of procedure the owners of the patent are granted the monopoly of the manufacture of the Morrison chair for another seventeen years, which is acquired by a patent applied for over twelve years ago; making a total of over twenty-nine years, a thing that was never intended by the framers of the laws governing the issuing of patents. Had we had a dental examiner in the Patent-Office, the matter would have been brought to light and the remedy applied. Let us have a representative of the dental profession in the Patent-Office whose duty it shall be to look out for the interests of the body he represents, and thus prevent the issuing of patents on articles which possess no real value, are devoid of novelty, are copies of nature, thereby presenting no *new* features, or are based upon methods of procedure which have long been in use.

